# The Role of Long Non-Coding RNAs in Thyroid Cancer

**DOI:** 10.3389/fonc.2020.00941

**Published:** 2020-06-11

**Authors:** Xuejiao Peng, Kun Zhang, Li Ma, Junfeng Xu, Weiqin Chang

**Affiliations:** ^1^Department of Thyroid Surgery, Second Affiliated Hospital of Jilin University, Changchun, China; ^2^Medical Research Center, Second Affiliated Hospital of Jilin University, Changchun, China

**Keywords:** thyroid cancer, molecular mechanism, biomarker, tumorigenesis, long non-coding RNAs

## Abstract

Thyroid cancer, the most common endocrine malignancy, has become the most commonly diagnosed malignant solid tumor. Moreover, some cases have poor prognosis, and the survival period is only 3–5 months. Long noncoding RNAs (lncRNAs) are a group of functional RNA molecules more than 200 nucleotides in length that lack the ability to encode protein but participate in all aspects of gene regulation. Functionally, many lncRNAs play essential roles in epigenetic regulation at transcriptional and post-transcriptional levels via various molecular mechanisms. Recent studies have discovered important roles for lncRNAs during the complex process of carcinogenesis in thyroid cancer. In this review, we focus on lncRNAs dysregulated in thyroid cancer and summarize recently reported associations between lncRNAs and thyroid cancer in order to demonstrate the significant value of lncRNAs in diagnosis and treatment.

## Introduction

Thyroid cancer is the most common endocrine tumor. Its incidence is reportedly seventh highest among female malignancies in developed countries and ninth highest among female malignancies in developing countries (1). In recent years, the incidence of thyroid cancer worldwide has increased significantly, which may be attributed to earlier screening. Age-standardized statistics show that the global incidence of thyroid cancer in 2016 was 2.2/100,000 men per year and 4.4/100,000 women per year, and the incidence increased by 50% from 2006 to 2016, the fastest increase for any malignant solid tumor (2). As a female-prone tumor, thyroid cancer ranks first among malignant tumors affecting the female population in various countries and regions. Research shows that by 2019, thyroid cancer will rank third among such tumors (3). Although most thyroid cancers can be effectively controlled by surgery, endocrine inhibition therapy, and iodine radiation, mortality associated with advanced thyroid cancer and iodine-refractory thyroid cancer has not decreased. Understanding the pathogenesis of thyroid cancer, and finding biomarkers for its early diagnosis and effective treatment are current focal points of research.

Studies have shown that only 2% of the genes of many mammals are involved in the protein translation process, while the remaining 98% of RNAs are only involved at the transcriptional level and are called “non-coding RNAs” (ncRNAs). Based on their molecular sizes, ncRNAs are classified as short ncRNAs or long ncRNAs (lncRNAs). Short ncRNAs include miRNAs, tRNAs, interfering small RNAs (siRNAs), RNAs that interact with Piwi proteins (PiRNAs), and certain ribosomal RNAs. lncRNAs have broader research prospects and are a current research focus. lncRNAs, as ncRNAs longer than 200 nt, are not translated to proteins due to their lack of open reading frames. Although lncRNAs are not involved in protein transcription, they can regulate gene expression at multiple levels and are related to tumorigenesis. In recent years, with the emergence of next-generation sequencing, third-generation sequencing, RNA-Seq, RIP-Seq, and RNA arrays, more and more lncRNAs have been discovered. Recent reports and ongoing studies have found that many lncRNAs are closely related to thyroid cancer. This article discusses the recent research progress on lncRNAs and papillary thyroid cancer (PTC) by reviewing the latest research reports.

## Functional Lncrnas Involved in Thyroid Cancer

An increasing number of studies have confirmed that differential expression of lncRNAs is closely related to the biological behavior of thyroid cancer. Yang et al. (4) compared the expression of lncRNAs in three groups of thyroid cancer tissues and paracancerous tissues by microarray analysis and found that there were 675 differentially expressed lncRNAs in PTC, of which 312 were upregulated and 363 downregulated. In addition, some studies have used certain lncRNAs to establish biomarker systems for detecting recurrence and prognosis in patients with PTC (5). However, the value of lncRNAs in the diagnosis, treatment, and prognosis of thyroid cancer warrants further research.

Thus far, many lncRNAs have been found to be closely associated with the occurrence and progression of thyroid cancer. Additionally, the studied mechanisms of action of lncRNAs in thyroid cancer include the following: lncRNA-miRNA-protein, lncRNA(-miRNA)-target genes, epigenetics, and signaling pathways. Representative lncRNAs include *NEAT1, HOTAIR, PTCSC2, lncRNA TNRC6C-AS1, GAS8-AS1, PTCSC3, MEG3, BANCR, PVT1, SPRY4-IT1, GAS5, H19, CASC2*, and *MALAT1*.

### lncRNA-miRNA-Protein

This is currently the most common mechanism of action associated with lncRNAs, which are regarded as among the most important competing endogenous RNAs (ceRNAs) and participate in the regulation of gene networks by acting on target genes or proteins. lncRNAs promote proliferation of thyroid cancer cells by competitively inhibiting miRNAs through regulation of downstream proteins highly expressed in thyroid cancer tissues.

#### Neat1

lncRNA-rich nuclear-rich transcript 1 (*NEAT1*), located on chromosome 11q13.1, was discovered by Hutchinson et al. (6). *NEAT1* reportedly acts as a ceRNA in tumorigenesis (7–12). There is increasing evidence that lncRNAs act as molecular sponges in many malignancies (13). *NEAT1* is overexpressed in thyroid cancer tissues and cells compared to levels in normal thyroid tissues and cells. Highly expressed *NEAT1* reportedly promotes β-catenin expression by interacting with miR-214. β-catenin is a direct target of miR-214 and participates in the malignant behavior of *NEAT1*-induced thyroid cancer (14). Zhang et al. (15) confirmed that *NEAT1* is upregulated in thyroid carcinoma, and that its upregulation can inhibit the action of miRNA-129-5p and upregulate kallikrein-related peptidase 7 (KLK7) expression. As the seventh member of the serine protease family, KLK is increasingly found to be overexpressed in human cancers and facilitates cancer metastasis through degradation of cell junction proteins (15). Its dysregulation is related to tumorigenesis in ovarian, breast and cervical cancers, and melanoma. Overexpression of KLK7 is also closely related to poor prognosis in thyroid cancer. *NEAT1* has two isoforms: *NEAT1_1* (3.7 kb) and *NEAT1_2* (23 kb). As an oncogene, *NEAT1_2* is upregulated in thyroid cancer and is linked to tumor size and TNM stage. Sun et al. (16) reported that *NEAT1_2* can regulate the expression of ATPase family AAA domain-containing 2 (ATAD2) by downregulating miR-106b-5p in papillary thyroid cancer. The study found that ATAD2 is abnormally expressed in hepatocellular carcinoma, prostate cancer, lung cancer, ovarian cancer, and cervical cancer, and its expression level is associated with tumor stage, histological grade, and lymph node metastasis (17). *NEAT-2*-targeted therapy may become a treatment option for thyroid cancer in the future.

Typically, postoperative radioactive iodine (RAI, 131I) treatment improves prognosis in some patients with thyroid cancer. However, Liu et al. (18) reported that *NEAT1* suppressed the expression of miR-101-3p to upregulate fibronectin 1 (FN1), and ultimately invalidated the effect of RAI treatment. Additionally, overexpression of FN1 promotes activation of the PI3K/AKT signaling pathway, leading to RAI resistance in PTC.

FN1, a basic component of the extracellular matrix, is a biomarker of the epithelial-mesenchymal transition (EMT), which is positively correlated with PTC lymph node metastasis (LNM) (19). Xia et al. (19) indicated that FN1 overexpression was associated with larger PTC tumor size, PTC LNM, and advanced pTNM stage, causing recurrence and affecting prognosis.

#### Hotair

HOX antisense intergenic RNA (*HOTAIR*) is a 2.2 kb RNA molecule expressed from the Hoxc cluster located in chromosome 12q13.3 and is among the best-studied lncRNAs in cancer. Overexpression of *HOTAIR* has been linked to poor prognosis and increased invasiveness in cancer (20), and to the invasion and migration of hepatocellular carcinoma and glioma (21, 22). Thus far, there have been relatively few studies on the relationship between *HOTAIR* and thyroid cancer. *HOTAIR* has been found to be significantly upregulated in thyroid carcinoma cells as well as thyroid cancer tissue samples and plasma. Moreover, higher expression levels of plasma HOTAIR were positively correlated with worse 5-year survival rates in patients with thyroid cancer (23). Additionally, *in vitro* experiments indicated that knocking down *HOTAIR* can significantly inhibit the growth and invasion of thyroid cancer cells. In a study by Di et al. (24), *HOTAIR* overexpression in thyroid cancer cells and tissue inhibited miRNA and cyclin D2 (CCND2) protein activation. Silencing HOTAIR expression inhibits thyroid cancer cell growth *in vivo* and *in vitro*. *HOTAIR* negatively regulates miR-1 by direct competitive binding to the miR-1 locus and participates in the regulation of thyroid cancer cell carcinogenesis. CCND2 belongs to the conserved cyclin family, which controls the cell cycle, and has been shown to be highly expressed in ovarian and testicular tumors. The *HOTAIR*/miR-1-CCND2 axis may be a new direction for lncRNA and thyroid cancer research. There are also other hypotheses about the relationship between *HOTAIR* and thyroid cancer. Zhu et al. (25) reported that the *HOTAIR* rs920778T allele, a PTC risk allele, is associated with significantly increased *HOTAIR* expression. Some studies have explored the relationship between lncRNA and thyroid cancer using bioinformatics, constructing ceRNA regulatory networks using the Gene Expression Omnibus (GEO) database and the Cancer Genome Atlas (TCGA). Chen et al. (26) hypothesized that *HOTAIR* may be involved in the development of PTC by interrupting neuronal growth. A study based on the TCGA and GEO databases showed that *HOTAIR* overexpression in PTC is linked to tumor size, lymph node metastasis, and poor prognosis, and may play an oncogenic role through the Wnt pathway (27). The mechanism linking PTC and *HOTAIR* warrants further study, as the expression of *HOTAIR* in plasma is being considered a novel diagnostic biomarker in PTC.

#### lncRNA TNRC6C-AS1

lncRNA *TNRC6C-AS1* acts as a ceRNA on miR-129-5p in thyroid cancer. Hou et al. (28) reported that *TNRC6C-AS1* sponges miR-129-5p and regulates UNC-5 netrin receptor B (UNC5B) in thyroid cancer, influencing cell proliferation, migration, and invasion. UNC5B is a netrin-1-dependent receptor that participates in axonal migration and angiogenesis by binding to netrin-1, which exerts its function in tumor suppression. The role of UNC5B in other tumors has been confirmed (29), but its role in thyroid cancer has rarely been studied. Studies have also found that *TNRC6C-AS1* is upregulated in PTC tissues and increases proliferation, migration, and invasion of TPC1 cells. The expression of *TNRC6C-AS1* was negatively correlated with mRNA levels of its coding partner, TNRC6C in PTC tissues. The study also found that knockdown of *TNRC6C-AS1* or overexpression of TNRC6C upregulated the expression of iodine metabolism genes, including *NIS, TPO, TSHR*, and pendrin (30). The *TNRC6C-AS1*-TNRC6C axis plays an important role in tumorigenesis, invasion, and iodine accumulation in PTC.

#### PTCSC2

Papillary thyroid cancer susceptibility candidate 2 (*PTCSC2)* is a 60-nucleotide lncRNA located on chromosome 9q22 (31) and is divided into two subtypes: folded and unfolded. A study involving 65 thyroid cancer tissues found that both folded and unfolded *PTCSC2* were expressed at lower levels than in normal thyroid tissues. The locus on chromosome 9q22 contains an SNP closely related to PTC risk (rs965513). The locus also includes the forkhead box E1 (*FOXE1*) gene associated with thyroid development and *PTCSC2* (32). *PTCSC2* may work synergistically with rs965513 to cause thyroid cancer. Another recent report found that myosin-9 (MYH9) acts as an lncRNA-binding protein that targets the FOXE1 promoter region through interaction with *PTCSC2* and exerts its regulatory function in thyroid cancer via downstream pathways; this may be the molecular mechanism by which the gene and rs965513 act (33). At present, there have been few studies on the role of *PTCSC2* in thyroid cancer, and more research is needed to determine their relationship.

### lncRNA(-miRNA)-Target Genes

Some lncRNAs act directly on target genes, while others act indirectly through miRNAs. Some lncRNAs act on target genes through both direct and indirect pathways.

#### GAS8-AS1

GAS8 antisense RNA 1 (*GAS8-AS1*) is located in the second intron of GAS8 and transcribes a 994 nt ncRNA in the opposite orientation of GAS8, which is reported to be a novel tumor suppressor that affects tumor cell proliferation in PTC. *GAS8-AS1* was first reported in PTC by Pan et al. (34), but its regulatory mechanism is still unclear. Using whole exome sequencing, Pan et al. reported that *GAS8-AS1* is the most frequently altered gene other than BRAF. Recently, lncRNA *GAS8-AS1* was further studied by Zhang et al. (35), who reported that its expression was dramatically lower in the plasma compared with levels observed in benign nodule controls and normal goiter. This study also associated downregulation of *GAS8-AS1* with LNM; thus *GAS8-AS1* can be used as a biomarker for LNM prediction. In addition, Qin et al. (36) found that the relative expression of *GAS8-AS1* was significantly reduced in four PTC cell lines compared to levels in normal thyroid cell lines; they also found that the role of *GAS8-AS1* in thyroid cancer may be related to autophagic activity. *ATG5* is a key autophagy-related gene (*ATG*) associated with malignancy. Overexpression of *GAS8-AS1* upregulates ATG5 at mRNA and protein levels. Yuan et al. found that *GAS8-AS1* affects autophagy and proliferation by regulating the expression of *ATG5*. Plasma levels of lncRNA *GAS8-AS1* may be a promising biomarker for the diagnosis, prognosis, and treatment of thyroid cancer.

#### PTCSC3

*PTCSC3* is an lncRNA on locus 14q13.3 and is highly thyroid-specific (37). Jendrzejewski et al. (37) performed an experimental study including 46 cases of PTC and paracancerous tissues and found that the expression of *PTCSC3* in PTC tissues was significantly lower than that in adjacent normal thyroid tissues. Another study found that rs944289 was significantly correlated with benign and malignant thyroid tumors in Japanese patients (38). Additionally, the size of the thyroid tumor and the extrathyroidal invasion of the tumor were significantly correlated with the expression level of rs965513. S100A4 belongs to a large family of EF-hand domain calcium-binding proteins and has become a recognized marker of cancer metastasis in S100 transcripts. Overexpression of the S100A4 gene was first associated with the occurrence, development, and prognosis of breast cancer, gastric cancer, rectal cancer, lung cancer, melanoma, and other cancers (39–43). The same gene was later confirmed to be closely related to thyroid cancers other than medullary cancer. Jendrzejewski et al. (44) found that decreased expression of *PTCSC3* in PTC tissue can promote the overexpression of the S100A4 gene and the proliferation, invasion, and metastasis of thyroid cancer cells. Donato (45) found that the expression levels of vascular endothelial growth factor (VEGF) and matrix metalloproteinase-9 (MMP-9) were also abnormal in cells with abnormal expression of S100A4. They found that the levels of VEGF and MMP-9 in BCPAP thyroid cancer cells expressing *PTCSC3* were significantly downregulated to inhibit invasion and metastasis. This mechanism of action may be that *PTCSC3* inhibits the secretion of VEGF and MMP-9 by inhibiting S100A4 expression, thereby inhibiting the invasion and metastasis of thyroid cancer cells.

#### MEG3

Maternal expressed gene 3 (*MEG3*) located on human chromosome 14q32 is usually expressed in many normal tissues. However, downregulation of *MEG3* expression is closely related to tumorigenesis. A decrease in *MEG3* expression has been found in many human tumors, including in the stomach, tongue, prostate, lung, and bladder, and it exerts its function in tumor cell proliferation, migration, and invasion (46–51). Wang et al. (52) reported that expression of *MEG3* is lower in metastatic tissues of PTC than that in non-metastatic thyroid cancer tissues. In human thyroid cancer cell lines, high levels of *MEG3* can inhibit invasion and metastasis of thyroid cancer cells. This study showed that the *Rac1* gene is negatively regulated by *MEG3* at the posttranscriptional level in thyroid cancer. *MEG3* inhibits migration and invasion of thyroid cancer cells by acting on Rac1 and is linked with lymph node metastasis. Rac1 is one of the most studied Rho GTPases. It exerts its regulatory function in cell proliferation, participates in the signaling pathway promoting cell survival, and plays a central role in the control of cell adhesion and migration. Additionally, Liu et al. (53) reported that *MEG3* inhibits proliferation of 131 I-resistant TC cells by negative regulation of miR-182, induces apoptosis, and enhances DNA damage. These results indicate that *MEG3* functions as a tumor suppressor, resulting in the inhibition of tumor growth in thyroid cancer.

### Epigenetic Modification

lncRNAs have been reported to directly bind target proteins and conduct post-transcriptional modification in many cancers (54–57). Histone modifications are a type of epigenetic modifications. A variety of lncRNAs are reportedly involved in the biological activities of thyroid cancer through histone modification.

#### BANCR

BRAF-activated ncRNA (*BANCR*), a 693 bp-long transcript on chromosome 9q21.12, was first discovered in 2012 by Flockhart et al., and is considered a potential regulator of melanoma cell metastasis (58). *BANCR* is strongly linked to BRAF V600E, the most prevalent mutation in thyroid cancer genes. Studies have shown that *BANCR* produced by the BRAF V600E mutation is also associated with the occurrence of thyroid tumors (59). *BANCR* has both carcinogenic and tumor suppressing effects, and can play different roles in different tumors. It has been reported that *BANCR* has a tumor suppressing effect in liver and bladder cancers (60, 61), while it acts as an oncogene in gastric, colorectal, and lung cancers (62–64).

Enhancer of zeste homolog 2 (EZH2), an oncogenic histone methyltransferase, is a well-known histone modifier. Overexpression of EZH2 has been strongly associated with several types of cancer (65, 66). Zheng et al. (67) found that the expression of *BANCR* in PTC tissues was significantly higher than that in adjacent tissues. The study showed that *BANCR* can be recruited by EZH2 to increase the expression level of thyroid-stimulating hormone receptor (TSHR) and promote the proliferation of IHH-4 thyroid cancer cells. TSH exerts its effects on thyroid cell proliferation by binding to its receptor, TSHR. By silencing *BANCR*, chromatin recruitment of EZH2 and expression of TSHR can be reduced. Zheng et al. (68) also reported that *BANCR* may promote the development of malignant thyroid nodules via the modulation of TSHR expression and its downstream effector, cyclin D1. However, Wang et al. found that *BANCR* promotes EMT in PTC cell lines by activating the Raf/MEK/ERK signaling pathway (69). Liu et al. (70) reported that *BANCR* affects the proliferation, invasion, and apoptosis of thyroid cancer cells through modulation of autophagy behavior. The expression of *BANCR* was positively related to the pathological stage of thyroid carcinoma and lymph node metastasis. In their study, Zhang et al. (71) showed that downregulation of *BANCR* promotes aggressiveness in PTC via the MAPK and PI3K pathways. These studies indicated that *BANCR* could function as both an oncogene and a tumor-suppressor gene; thus, whether *BANCR* is bifunctional in PTC warrants further clarifying investigation.

#### PVT1

lncRNA *PVT1*, located at chromosome 8q24.21, is highly expressed in many tumors. In a previous study, *PVT1* was reported to promote the proliferation of thyroid carcinoma cells by recruiting EZH2 and mediating TSHR expression (72). Recently, Feng et al. (73) found that *PVT1* is highly expressed in thyroid cancer tissues and cells, and its expression level is associated with TNM stage and lymph node metastasis in thyroid cancer. The expression level of *PVT1* in patients with lymph node metastasis and tumor infiltration presented with significantly higher expression level of *PVT1* than their counterparts without these aggressive disease features. lncRNA *PVT1*, as a ceRNA of microRNA-30a, was proven to enhance the invasion and metastasis of PTC cells by mediating expression of insulin-like growth factor 1 receptor (IGF1R). IGF1R exerts its function in maintaining homeostasis as well as normal thyroid morphogenesis, and papillary thyroid hyperplasia is promoted when certain IGF1R signals are lost. MiR-30a inhibits cell invasion, migration potential, EMT, and metastatic potential by binding and negatively regulating expression of its target gene, lysyl oxidase (*LOX*), which is associated with higher mortality in undifferentiated thyroid carcinoma. The association between *PVT1* and thyroid cancer remains to be studied further.

### Signaling Pathways

#### TGF-β Signaling Pathway

##### LncRNA SPRY4-IT1

*SPRY4-IT1*, a 708 bp lncRNA discovered by Khaitan et al. in 2011, is derived from an intron of the *SPRY4* gene residing on chromosome 5q31.3. Studies have confirmed that dysregulation of *SPRY4-IT1* is related to many cancers, including osteosarcoma, breast cancer, lung cancer, hepatocellular carcinoma, colorectal cancer, pancreatic cancer, and others (74–79). Zhou et al. (80) found that *SPRY4-IT1* was upregulated in thyroid cancer tissues and cell lines, and its high levels were strongly correlated with lymph node metastasis, clinical stage, and poor prognosis of patients with thyroid cancer (80). Additionally, *SPRY4-IT1* participates in the progress of thyroid cancer by regulating the TGF-β/Smad signaling pathway, which may provide a new perspective for studies on thyroid cancer and *SPRY4-IT1*.

#### PI3K/AKT Signaling Pathway

##### GAS5

The gene coding for lncRNA growth arrest-specific 5 (*GAS5*) is ~630 nt long and is located on chromosome 1q25. *GAS5* is associated with a range of malignancies, including rectal cancer, cervical cancer, glioma, and oral squamous cell carcinoma (81–84). It was first reported by Abudoureyimu et al. (85) in thyroid cancer, in which *GAS* expression reportedly decreases. Recently, Guo et al. (86) reported that *GAS5* expression was lower in thyroid cancer tissues than in benign tumor tissues. Low GAS5 expression is related to TNM staging, lymph node metastasis, multiple cancer foci, and poor prognosis in patients with thyroid cancer. Zhang et al. (87) found that *GAS5* is downregulated in thyroid cancer tissues and thyroid cancer cell lines. This study showed that *GAS5*, as a ceRNA, acts on miR-222-3p in thyroid cancer, leading to activation of the *PTEN*/*AKT* pathway and exerting an anti-cancer effect. *PTEN* is a key inhibitory gene for tumor cell growth that inhibits AKT phosphorylation, and its downregulation is related to tumorigenesis. Therefore, *GAS5* may be a potential prognostic marker and therapeutic target in PTC.

##### H19

lncRNA- *H19* is located on human chromosome 11p15.5 and is involved in a great many cancers, including lung cancer, breast cancer, and gastric cancer, among others. One study found that *H19* plays a more complex role in tumorigenesis, both carcinogenic and tumor suppressive. Thus far, few studies have examined the relationship between *H19* and thyroid cancer. Lan et al. (88) found that decreased expression of *H19* in PTC is closely related to lymph node metastasis. In the future, *H19* may be used to optimize management in patients with uncertain fine-needle aspiration specimens. In addition, it could be used as a potential tool to distinguish patients with and without lymph node metastasis. Liu et al. (31) and Jiao et al. (89) reported that increased H19 expression levels are associated with tumor diameter, TNM stage, lymph node metastasis, and poor prognosis in TC patients. Wang et al. (90) reported that *H19* can inhibit cell proliferation by downregulating IRS-1 in thyroid cancer cells. IRS1 regulates the activation of the PI3K/AKT and nuclear factor κ-β signaling pathways. The PI3K/AKT pathway exerts its function in tumorigenesis. It has been shown to promote malignant transformation of tumors by enhancing cancer cell survival, proliferation, and metabolism (91). Li et al. (92) also found that *H19* plays an oncogenic role in thyroid cancer through the PI3K/AKT pathway. However, Liu et al. (93) found that *H19* acts as an oncogene in thyroid cancer. *H19* can promote invasion and metastasis of thyroid cancer cells *in vivo* and *in vitro*. As a ceRNA, lncRNA antagonizes the function of *H19*/miR-17-5p and upregulates the expression of their target YES1, inducing cell cycle progression. Knocking down *H19* can inhibit cell proliferation *in vivo* and *in vitro*. YES1 belongs to the protein tyrosine kinase family and is upregulated in many cancers. In addition, the study linked *H19* overexpression to poor prognosis in patients with thyroid cancer (93).

#### EMT Pathway

##### CASC2

lncRNA cancer susceptibility candidate 2 (*CASC2*), located on chromosome 10q26, has been associated with a variety of malignancies. However, studies on the association between *CASC2* and thyroid cancer are rare. Xiong et al. (94) reported that *CASC2* is downregulated in thyroid cancer tissues, and its overexpression *in vitro* can inhibit the proliferation of thyroid cancer cells by interfering with the cell cycle. Downregulation of *CASC2* expression was significantly associated with multifocality and TNM staging of tumors, but not with other clinical pathological parameters. *CASC2* downregulation indicates poor prognosis in thyroid cancer. Recently, *CASC2* has been reported to be significantly downregulated in tissue and plasma samples from patients with PTC compared to levels in patients with nodular goiter, and its expression level is significantly related to LNM (95). In addition, this study demonstrated that *CASC2* affects thyroid cancer cell invasion and metastasis by regulating the EMT pathway, and may be a predictor of LNM in patients with thyroid cancer. Compared with that in normal tissues, the expression of *CASC2* was significantly decreased in PTC tumors, and the downregulation of *CASC2* was significantly associated with tumor size, presence of multifocal lesions, and advanced pathological stage. Overexpression of *CASC2* leads to inactivation of AKT and ERK1/2, which can significantly inhibit the proliferation of thyroid cancer cells (96).

##### MALAT1

Metastasis-associated lung adenocarcinoma transcript 1 (*MALAT1*) is a lncRNA of more than 8.0 kb in length, and its gene is located at 11q13.1. *MALAT1* is associated with many cancers (97–99). Studies have found that *MALAT1* can promote the proliferation and metastasis of thyroid cancer cells (100, 101). In a previous study, Huang et al. (102) examined 10 cases of follicular thyroid carcinoma tissue and 10 cases of normal thyroid tissue and found that *MALAT1* was upregulated in thyroid cancer tissues. *MALAT1* promotes the formation of blood vessels in thyroid cancer by regulating the secretion of the FGF2 protein in tumor-associated macrophages (TAMs), thereby promoting biological behaviors such as proliferation and metastasis in thyroid cancer cells. Chu et al. (101) reported that the expression level of *MALAT1* was significantly upregulated in medullary thyroid carcinoma compared with that in normal thyroid tissue. Furthermore, *in vitro* experiments have shown that inhibition of *MALAT1* exerts an anti-tumor effect and inhibits cell proliferation and invasion. Zhang et al. (103) found that *MALAT1* expression was upregulated in PTC TPC1 cells induced by transforming growth factor (TGF)-β to the EMT, which provided a new perspective for lncRNA research. This study also found that *MALAT1* expression levels in poorly differentiated thyroid carcinomas and anaplastic thyroid carcinomas are significantly lower than those in normal thyroid tissues, with expression in anaplastic thyroid carcinomas (ATCs) showing the lowest levels. This was the first report of *MALAT1* being downregulated in any malignancy, and it indicates that *MALAT1* may be a potential tool in classification of thyroid carcinoma. Recently, Liu et al. (104) found that *MALAT1* expression was upregulated in PTC tissues, and the upregulated *MALAT1* expression was correlated with tumor size, lymph node metastases, and disease stage. Wen et al. (105) explored the potential correlation between *MALAT1* genetic variations (single nucleotide polymorphism; SNP) and the risk of PTC. They found that *MALAT1* SNP rs619586 could directly reduce *MALAT1* expression, becoming a potential protective factor that reduces the risk of PTC. *MALAT1* may play different roles in different thyroid tumors, and its effect is determined by the type of thyroid cancer. Gene polymorphisms may be a potential focal point in study of the relationship between lncRNA and PTC.

#### Wnt/β-catenin Signaling Pathway

Certain lncRNAs function in a variety of ways. *PTCSC3* can act directly on its target gene S100A4 or through a signaling pathway. Xia et al. (106) found that *PTCSC3* reduces the proliferation and invasion of glioma cells by inhibiting the action of the Wnt/β-catenin signaling pathway. Additionally, Wang et al. (107) reported that PTCSC3/miR574-5p promotes proliferation and migration of papillary thyroid carcinoma cells via the Wnt/β-catenin signaling pathway.

The lncRNAs discussed above and listed in Table 1 are likely to function in thyroid cancer.

**Table 1:** LncRNAs are related to thyroid cancer.

**No**	**Approved symbol**	**lncRNA name**	**Gene locus**	**Expression level in patient**	**Molecules and pathways interacting with lncRNA in thyroid cancer**	**References**
1	BANCR	BRAF-activated non-coding RNA	Chr. 9q21.12	Overexpression	EZH2, EMT, the Raf/MEK/ERK signaling pathway	(67, 69)
2	PTCSC2	Papillary thyroid carcinoma susceptibility candidate 2	Chr.9q22	Underexpression	MYH9	(33)
3	PVT1	PVT1 oncogene	Chr.8q24.21	Overexpression	Insulin like growth factor 1 receptor, EZH2, microRNA-30a	(72, 73)
4	NEAT1	Nuclear-enriched abundant transcript 1	Chr.11q13.1	Overexpression	miR-129-5p, miR-214	(14, 15)
5	HOTAIR	Hox transcript antisense intergenic RNA	Chr.12q13.3	Overexpression	Wnt pathway, CCND2	(24, 25)
6	TNRC6C-AS1	TNRC6C antisense transcript 1		Overexpression	UNC5B, TNRC6C	(28)
7	MEG3	Maternally expressed gene 3	Chr.14q32.2	Underexpression	miR-182, Rac1	(52, 53)
8	GAS8-AS1			Underexpression	ATGs	(36)
9	PTCSC3	Papillary thyroid carcinoma susceptibility candidate 3	chr.14q13.3	Underexpression	S100A4 protein, miR574-5p, Wnt/β-Catenin Signaling	(44, 45)
10	SPRY4-IT1	SPRY4 intronic transcript 1	Chr.5q31.3	Overexpression	The-receptor-transduced mitogen-activated protein kinase pathway	(80)
11	GAS5	Growth arrest specific 5	Chr.1q25.1	Underexpression	miR-222-3p	(86, 87)
12	H19	H19	Chr.15p.15.5	Overexpression	miR-17-5p, TNFR2	(90, 93)
13	CASC2	Cancer susceptibility candidate 2	Chr.10q26	Underexpression	EMT	(95, 96)
14	MALAT1	Metastasis-associated lung adenocarcinoma transcript 1	Chr.11q13.1	Overexpression	EMT, TAM	(102, 103)

## The Promising Future of lncRNAs in Cancer Diagnosis and Prognosis

Many studies have found that certain lncRNAs are stably present in human serum/plasma (108, 109), which may be helpful in the study of the role of serum/plasma lncRNAs in diagnosis and prognosis. Circulating markers have been widely used for disease prediction. Shi et al. (109) reported that the expression of lncRNAs in body fluids and serum/plasma have significant value in the diagnosis of many cancers. Zhou et al. (110) reported that the expression of *H19* in plasma has high specificity and sensitivity for diagnosis of gastric cancer and was more effective than conventional biomarkers, such as carcinoembryonic antigen (CEA) and carbohydrate antigen 199 (CA199). Tang et al. (111) found that expression of lncRNAs in saliva is associated with the prognosis of oral squamous cell carcinoma and can provide clinics with a non-invasive and convenient screening tool. To date, many circulating lncRNAs have been shown to have diagnostic significance in thyroid cancer, including *HOTAIR, H19, MALAT1, GAS5, GAS8-AS1, DLG1-AS1, ENST00000462717, ENST00000415582, TCONS_00024700*, and *NR_028494* (34, 80, 102, 112). The latter four are considered linked to prognosis of patients with PTC and lung metastases (113). Zhang et al. (35) identified plasma *GAS8-AS1* overexpression in the serum of patients with thyroid cancer. He et al. (112) found that plasma *DLG1-AS1* was upregulated in patients with PTC but not in patients with benign thyroid nodules or healthy controls. Plasma testing for biomarkers not only enables early diagnosis, avoiding poor prognoses, but also prevents unnecessary treatments. Circulating lncRNAs may be potential tumor markers for PTC diagnosis.

Thus far, many lncRNAs have been associated with PTC. Most patients with PTC have a favorable prognosis with the current therapeutic regimen, which includes surgical resection, thyroid hormone suppression, and radioactive iodine therapy. However, a small proportion of PTC cases have poor prognoses due to metastases. Therefore, it is critical to differentiate these patients from lower risk cases at the early stage. Screening for some lncRNAs could contribute to molecular stratification of aggressive and indolent PTC and accumulating evidence has implicated lncRNAs in PTC LNM. Studies carried out via microarray, TCGA PTC cohorts, or ceRNA networks have found that many previously uncharacterized lncRNAs are associated with poor prognosis or LNM. Song et al. (114) found that high *ENS-653* expression is associated with more advanced tumor stage and poorer disease-free survival. *RP11-547D24.1* and *UNC5B-AS1* could differentiate patients with different stages of PTC, and some lncRNAs play vital roles in determining histological cancer type (115). Additionally, some lncRNAs are associated with LNM (116).

Although the expression of lncRNAs in serum is meaningful for studying certain biological behaviors of thyroid cancer, the measurement of lncRNAs in serum is affected by many factors. Studies have found that lncRNAs contained in blood cells may affect the measurement of lncRNAs in serum (117). The release of lncRNAs from blood cells during coagulation can cause the concentration of lncRNAs in serum to be higher than that in plasma. Factors such as diet and environment of the subjects also affect serum lncRNA concentrations. Food is a key confounding factor, and it is difficult for lncRNAs from food to be distinguished from endogenous lncRNAs once the former enter the circulation (118, 119). There are other factors that may affect circulating lncRNAs. The use of lncRNAs as molecular markers for clinical diagnosis may present significant challenges.

Thus far, several molecular markers, including *RAS, RET-PTC*, and *BRAF* (V600E) gene mutations, have been linked to PTC. BRAF mutations are the most common. According to the exome and RNA sequences, proteomic profiles, and epigenetic changes, Agrawal et al. (120) subdivided PTCs into BRAF-like and RAS-like groups. The RET (+) PTCs were much closer to BRAF-like PTCs than to RAS-like ones. A study reported that RAS mutations are more frequent in poorly differentiated thyroid carcinomas and anaplastic ones, but are rare in PTCs (121). *BRAF* (V600E) is a thyroid cancer-specific gene, and many studies have showed a significant association between the BRAFV600E mutation in PTC and factors characteristic of poor prognosis. Studies found that there are relationships between lncRNAs in PTC and the most common genetic alterations in PTC. Heejei Yoon et al. (122) reported that *NAMA* underexpression correlates with BRAF mutation. Wang et al. (123) reported that BRAF mutation is associated with the over-expression of many oncogenic molecules in PTC, including CCND1, CDKN1A, PERP, THBS1, and ZMAT3. Further, patients with BRAFV600E mutation had a higher expression of *ENS-653* (114). *COMET*, a new natural antisense lncRNA, maps on chromosome 7q31.2 and is highly expressed in BRAF-like carcinomas (124). COMET is a MET regulator and has been identified as a new MAPK-induced cytosolic lncRNA. Rusinek et al. (125) identified 18 BRAF-induced genes that are specific for BRAF V600E-driven PTC and seven BRAF-induced genes had not been previously reported as being related to BRAF mutation or thyroid carcinoma: *MMD, ITPR3, AACS, LAD1, PVRL3, ALDH3B1*, and *RASA1*. These results reported the influence of the BRAFV600E mutation on early PTC gene expression profile. Additionally, the expression of some lncRNAs are associated with BRAF (V600E) mutation. These lncRNAs may be associated with poor prognosis in PTC; thus, lncRNAs with subtype-specific expression stratified by BRAF mutation might be significant in individual molecular subtypes.

Related treatments targeting lncRNAs are under development, including nucleic acid-based therapies and plasmid-based therapies. At least 25 RNAi-based drug candidates are under clinical evaluation (126). Moreover, plasmid-based therapies are also being used in clinical phase III trials of bladder cancer (127). The idea that lncRNAs may be therapeutic targets in thyroid cancer has been proposed, and targeted therapies may be under development (31).

## Conclusion and Perspective

As molecular markers for tumor diagnosis and treatment, lncRNAs have been shown to participate in the proliferation, invasion, and metastasis of various malignant cells. In thyroid cancer, lncRNAs are potential biomarkers that could be used in diagnosis and in predicting invasion and metastasis. It has been reported that lncRNAs can be detected in plasma, serum, and other liquids with good stability and easy detection, suggesting new avenues for research. However, the regulatory mechanisms of lncRNAs in thyroid cancer remain to be further explored. There are still many difficulties in the extraction of lncRNA and future clinical applications. For example, under existing storage conditions, it is difficult to protect samples from RNA degradation, and the lncRNA database is relatively imperfect.

lncRNA is expected to become a target in gene-targeted therapy. Cancer treatment can be achieved by silencing or knocking down certain oncogenes. With the development of precision medicine, genetic diagnosis and treatment will become the trend, but there are still great challenges in research into lncRNA.

## Author Contributions

XP provided direction. XP, JX, and LM wrote the manuscript. WC and KZ made significant revisions to the manuscript. All authors read and approved the final manuscript.

## Conflict of Interest

The authors declare that the research was conducted in the absence of any commercial or financial relationships that could be construed as a potential conflict of interest.

## Abbreviations

ATAD2: ATPase family AAA domain-containing 2
CCND2: cyclin D2
ceRNA: competing endogenous RNA
EMT: epithelial to mesenchymal transition
EZH2: zeste homolog 2
FN1: fibronectin 1
FNA: fine-needle aspiration
FOXE1: forkhead box E1
GEO: Gene Expression Omnibus database
IGF1R: growth factor 1 receptor
IRS1: insulin receptor substrate 1
lncRNA: long non-coding RNA
LNM: lymph node metastasis
MYH9: myosin-9
PI3k/AKT: phosphatidylinositol 3-kinase/AKT
PTC: papillary thyroid cancer;
Rac1: rho GTPase1
SNP: single nucleotide polymorphism
TAM: tumor-associated macrophages
TCGA: The Cancer Genome Atlas
TGF: transforming growth factor
TSH: thyroid-stimulating hormone
TSHR: thyroid-stimulating hormone receptor
UNC5B: UNC-5 netrin Receptor B
VEGF: vascular endothelial growth factor.
